# The splenic Littoral cell angioma in China: a case report and review

**DOI:** 10.1186/1477-7819-9-168

**Published:** 2011-12-15

**Authors:** Zong-Qiang Hu, Yong-Jun A, Qiang-Ming Sun, Wen Li, Li Li

**Affiliations:** 1 Department of Hepatic-Biliary-Pancreatic Diseases, Second Affiliated Hospital of Kunming Medical University, Kunming 650031, China; 2 Molecular Epidemiology Joint Laboratory, Institute of Medical Biology, Chinese Academy of Medical Sciences, Kunming 650118, China; 3 Ganmei Affiliated Hospital of Kunming Medical University, Kunming 650011, China

**Keywords:** littoral cell angioma, vascular neoplasm, histology and immunohistochemistry, surgery

## Abstract

Littoral cell angioma (LCA) is a rare splenic vascular neoplasm that arises from the cells lining the red pulp sinuses. It is deemed to be a benign and incidental lesion. The earliest literature report of littoral cell angioma has been described by Falk. The examination of samples after splenectomy reveals similar pathological change and its change rule is summarized. However, many recent reports have described it to be a malignant tumor with congenital and immunological associations. Generally speaking, the definitive diagnosis can only be made after histological and immunohistochemical profiles. In this case report, we presented the case of a 48-year-old woman with multiple splenic LCAs. Initially, the patient was characteristics of abdominal distension, weakness and fatigue. Multiple hemangiomas were observed in the spleen through abdominal ultrasonic diagnosis. Computed tomography (CT) scans revealed the splenomegaly with multiple round and hyperdense lesions. The patient subsequently underwent splenectomy. Postoperative histological and immunohistochemical studies confirmed the diagnosis of LCA. Based on the presentation of this case, clinical, radiographic and pathological results of LCA as well as recent advances in our understanding of this uncommon splenic lesion were reviewed. LCA is an uncommon splenic tumor diagnosed in patients with or without abdominal discomfort. Only a few case reports regarding this kind of tumor have been published as inconsistent results. In the present paper, we have reported a case of LCA and reviewed the literature.

## Background

LCA is a benign neoplasm of the spleen that was first described by Falk et al. in 1991 [1,2], when they illustrated 17 cases of "a novel type of vascular tumor" [3]. Since then, no more than 80 additional cases have been reported [4,5]. The clinical representation of LCA ranges from being completely asymptomatic and discovered incidentally, to presenting with aconstellation of signs and symptoms such as abdominal distension, complex constitutional symptoms, splenomegaly, and hypersplenism [6,7]. Although the computed tomography (CT) and ultrasound (US) features of this neoplasm have been well described, there is a lack of specificity in differentiating the tumor from other primary vascular splenic tumors, namely, hemangiomas and angiosarcomas [8-10]. The definitive diagnosis can only be made after histological and immunohistochemical studies [11]. Since this is initial description, there only have scattered case reports and few case series of LCA. In this article, we would like to report a case of the rare splenic vascular neoplasm.

## Case report

**Figure 1:**
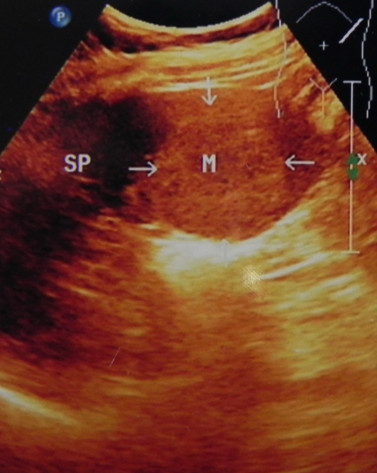
**Ultrasound of the spleen**. Ultrasound of the spleen demonstrates a well-defined hyperechoic lesion.

**Figure 2:**
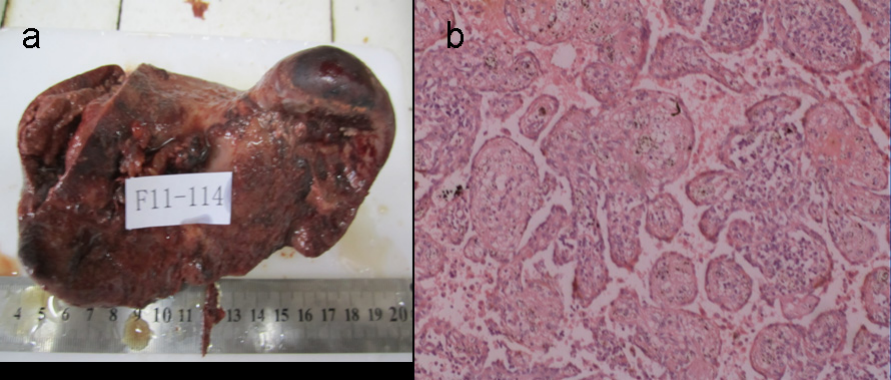
**The spleen**. Figure 2a:The spleen was 230.0 g in weight and 16.0 × 10.0 × 6.0 cm in dimension. Figure 2b: Higher-power view of the lesion demonstrates the characteristic histologic appearance of littoral cell angioma. The vascular spaces are lined with plump endothelial cells with the appearance of sinus lining, some of which have sloughed into the lumen (hematoxylin-eosin, original magnification 100×).

A 48-year-old Chinese woman was admitted due to non-specific complaints of abdominal distension, weakness and fatigue. This patient had been diagnosed with an oophoritic cyst 11 years ago and right oophorocystectomy. Meanwhile, this patient did not have medical history of weight loss, fever, or changes in bowel habits. Physical examination was notable for only splenomegaly. Abdominal ultrasonographic examination showed the increase in volume and multifocal processes throughout the spleen (Figure 1). CT scans confirmed the splenomegaly with multiple round and hyperdense lesions in the spleen. Contrast enhancement revealed an early hypoattenuation on arterial and most early portal phase scans. There was heterogeneous to homogeneous enhancement on late portal phase and delayed images. Some delayed scans have described a complete contrast washout with return to isoattenuation. In general, the CT can show an isoattenuated mass within the superior aspect of the spleen, which is barely perceptible on the noncontrast examination. The results from laboratory examinations were normal and the serologic examination for hepatitis A, B and C was negative. Our preoperative diagnosis was hemangioma. Subsequently, the patient underwent splenectomy. Post-operative rough pathological examination revealed a moderately enlarged spleen with approximately 1020 g in weight and 18 × 10 × 6 cm in dimensions. The capsular surface of the spleen showed multifocal sponge-like vascular spaces (Figure 2). The nodules with dark bluish red, smooth and multilobulated surface were ranged from 0.3 to 4.4 cm. In addition, these nodules were characterized as having multiple cystic spaces and structures resembling exaggerated red pulp sinusoids [12-14]. Histologically, this lesion was described as a vascular neoplasm with anastomosing vascular channels lined by histiocytes with occasional papillary structures, which was consistent with LCA (Figure 3). Immunohistochemistry was positive for factors CD31 and CD68 and negative for CD34 and CD21, thus confirming the diagnosis of LCA, and mitigating the diagnosis of angiosarcoma. The patient's post-operative course was uneventful, and her general condition was improved markedly within 100 days after the operation.

## Discussion

The differential diagnosis of splenic vascular tumors are broad and may represent benign (haemangioma, haemartoma and lymphangioma), indeterminate (LCA, haemangioendothelioma and haemangiopericytoma), or malignant neoplasm (angiosarcoma) [15]. Since the 1930s, endothelial cells lining the vascular sinuses of the spleen were considered as unique due to the exhibition of both phagocytic and hematopoietic properties [16]. Neoplasia of these cells results in the formation of LCA, which exhibits histological and molecular features consistent with both these epithelial and histiocytic cell types [17,18].

The exact incidence of LCA is unknown although the incidence of splenic haemangioma varies from 0.03% to as high as 14% in a series of autopsy reports [19]. Clinically, most patients (> 55%) are asymptomatic. The remaining patients with LCA present with splenomegaly, thrombocytopenia, anemia, or constitutional symptoms such as the fever of unknown origins [20]. The symptoms of anemia and thrombocytopenia may be hypersplenism-associated. Patients may also present with abdominal pain, or the tumor is diagnosed as an incidental finding [21].

Usually, the diagnosis is made after splenectomy for some other reasons. However, approximately 45% patients will present with splenomegaly, fever and features of hypersplenism (anaemia and thrombocytopenia) [22].

LCA may occur at any ages without sex-based predilection. Clinically, the disease may manifest as splenomegaly, anemia, and, less frequently, thrombocytopenia. LCA may also appear as a single or multiple lesions in the spleen. Diagnosis is usually made incidentally during surgery. Massive splenomegaly because of LCA can be mistaken as a pancreatic tumor.

Recent reports describe LCA as being associated with neoplasms of the colon, kidney, pancreas, lung and ovary [23,24]. Associations with leiomyosarcoma, melanoma and lymphoma have also been reported. In view of these findings, visceral neoplasm should be ruled out in all LCA patients.

## Conclusion

As a result of its unique histological and immunohistochemical characteristics, the definitive diagnosis can only be made on tissue samples obtained either cytologically or post-splenectomy because radiological findings are not specific for LCA. Diagnosis can be made by fine-needle biopsy, but surgery seems crucial because of suspected malignancy. Therefore, an evaluation for concomitant malignancy and surveillance is recommended.

## Consent

Written informed consent was obtained from the patient for publication of this Case report and any accompanying images. A copy of the written consent is available for review by the Editor-in-Chief of this journal.

## Competing interests

The authors declare that they have no competing interests.

## Authors' contributions

ZQH performed the literature review and manuscript writing. YJA reviewed and revised the manuscript and provided radiographic images. QMS reviewed and revised the manuscript and provided pathological images. WL provided original idea and assisted the manuscript revision. All authors read and approved the final manuscript.
